# An Optical Wireless Communication System for Physiological Data Transmission in Small Animals

**DOI:** 10.3390/s25010138

**Published:** 2024-12-29

**Authors:** Ana R. Domingues, Diogo Pereira, Manuel F. Silva, Sara Pimenta, José H. Correia

**Affiliations:** 1 CMEMS-UMinho, University of Minho, 4800-058 Guimarães, Portugal; anadomingues53@gmail.com (A.R.D.); diogopereira_07@hotmail.com (D.P.); fsilva@dei.uminho.pt (M.F.S.); sara.pimenta@dei.uminho.pt (S.P.); 2 LABBELS-Associate Laboratory, 4800-122 Braga, Portugal

**Keywords:** data transmission, optical telemetry, tissue phantom

## Abstract

In biomedical research, telemetry is used to take automated physiological measurements wirelessly from animals, as it reduces their stress and allows recordings for large data collection over long periods. The ability to transmit high-throughput data from an in-body device (e.g., implantable systems, endoscopic capsules) to external devices can also be achieved by radiofrequency (RF), a standard wireless communication procedure. However, wireless in-body RF devices do not exceed a transmission speed of 2 Mbit/s, as signal absorption increases dramatically with tissue thickness and at higher frequencies. This paper presents the design of an optical wireless communication system (OWCS) for neural probes with an optical transmitter, sending out physiological data through an optical signal that is detected by an optical receiver. The optical receiver position is controlled by a tracking system of the small animal position, based on a cage with a piezoelectric floor. To validate the concept, an OWCS based on a wavelength of 850 nm for a data transfer of 5 Mbit/s, with an optical power of 55 mW, was demonstrated for a tissue thickness of approximately 10 mm, measured in an optical tissue phantom.

## 1. Introduction

The ability to transmit data from in-body devices (e.g., implantable systems) for in vivo physiological measurements in small animals is a complex task. In wireless transmission for implantable medical devices, biological tissue significantly attenuates wireless signals. Moreover, this attenuation and the scattering of the signal are two characteristics to be considered to set the best interface [1].

Frequently, a large data collection must be transmitted (e.g., EEG signals or images) for long periods, and some conditions must be avoided: interferences when multiple animals are being used in a study, animal stress response and the effects of human handling [2].

Wireless communication can be established by resorting to low-power approaches, such as radiofrequency (RF), inductively coupled data transfer, capacitive coupled body channel communication, infrared optical communication, and ultrasonic communication [3].

RF wireless communication is generally used for a variety of applications, e.g., neuronal [4], cardio-respiratory function [5], and even automotive applications [6].

However, RF data rates for in-body devices typically remain below 2 Mbit/s, due to biological and regulatory constraints. Using the Shannon-Hartley theorem [7], with the FDA-allowed 344 MHz bandwidth [8] and the body’s typical signal-to-noise ratio (SNR) near −23 dB, practical rates are limited. Additionally, factors like ANSI C95.1-1982 power restrictions [9] and the rapid signal absorption in tissues at higher frequencies further reduce achievable rates [10]. These limits ensure safe penetration and operation within biological environments while adhering to FDA and ANSI standards.

Studies exist on the propagation of modulated infrared light in human tissue, and the theoretical achievement of up to 40 Mbit/s is described in the literature [11]. Moreover, light with a wavelength of 700–900 nm can pass through the skin to a depth of 3 mm. Additionally, infrared light with wavelengths below 800 nm can be easily transmitted by water, which is the main element of the human body. However, hemoglobin, the main element of blood, absorbs more infrared light [12].

To establish an optical link, specifically infrared optical communication, it is necessary to consider an optical transmitter (e.g., light-emitting diode (LED)) and an optical receiver (e.g., photodiode).

Different materials are used to define the emission wavelength of light-emitting diodes (LEDs) for designing the semiconductor (P–N junction) and its energy bandgap. The materials used to design LEDs with different wavelengths are summarized in Table 1 [13]. Thus, the most appropriate LED material for near-infrared (NIR) optical communications is aluminum gallium arsenide (AlGaAs).

Concerning the most appropriate photodiode for infrared optical communications, the standard silicon (Si) photodiodes are a good option for NIR wavelengths (up to approximately 1100 nm) [14].

**Figure 1:**
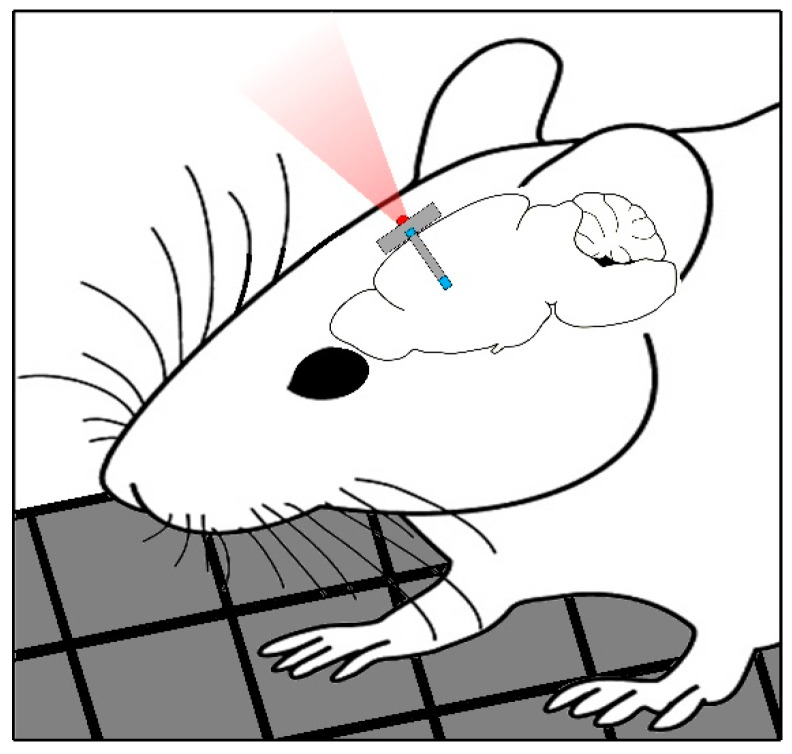
Schematic of an in-body neural probe in small animals with optical telemetry feature.

The ultimate goal of this work is the design of an optical wireless communication system (OWCS) based on NIR light, centered at 850 nm, that offers some advantages in comparison to the RF telemetry: (i) free from electromagnetic interference, (ii) it does not require an antenna, (iii) system miniaturization is possible, and (iv) large bandwidth. The selected wavelength presents favorable optical properties in terms of absorption and light scattering [15]. The OWCS is an alternative solution for wireless communication in neural probes (see Figure 1) with high-speed data transmission and the advantages previously described.

**Figure 2:**
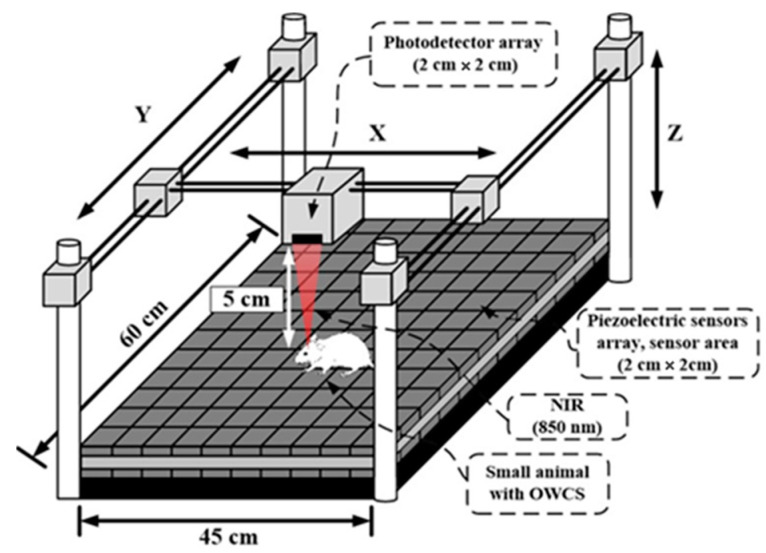
Animal cage with piezoelectric floor for tracking small animal position.

Additionally, we propose the design of a tracking system for the small animal based on a cage with a piezoelectric floor (see Figure 2), ensuring the alignment between the optical transmitter and optical receiver, which is required for a reliable optical link.

The use of OWCS requires the tracking of the optical beam from the transmitter to be detected by the optical receiver (a very low-cost solution compared with the complete active optical ceiling of the cage). The test cage for animal experimentation must reduce the amount of human effort required for data collection and allow for more natural, hands-off measurements in behaving animals. An animal cage (shown in Figure 2) was designed for recording small animals’ physiological signals in a free-behaving method. The cage with piezoelectric floor for tracking (CPFT) is a system that allows automatic tracking of an optical transmitter implanted in the brain of a small animal for neural signals at high transmission data rates.

**Figure 3:**
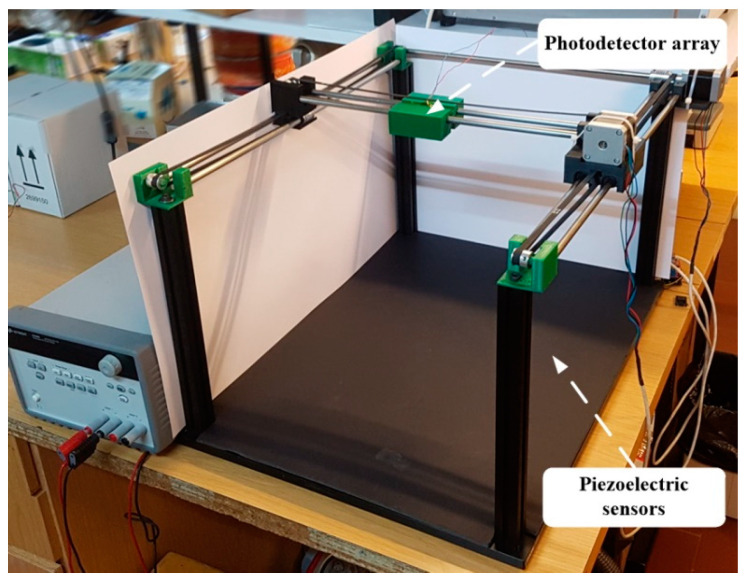
Real concept of the CPFT.

The animal cage floor is composed of a low-cost polyvinylidene fluoride (PVDF), a piezoelectric material, board divided into square pressure sensors for sensing motion. PVDF film sensors have advantages such as high flexibility, lightness, and high mechanical strength [16]. These polymer-based pressure sensors play a key role in detecting lightweight and providing precious information for a long time, exhibiting potential applications for real-time monitoring of movements (i.e., hopping, running, and walking) and gait [17]. The PVDF board has 60 cm by 45 cm of active area, being composed of individual 2 cm by 2 cm PVDF sensors, resulting in an array of 675 sensors. When a free-behavior animal presses one or more sensors, a certain pressure is applied to the sensor, and the PVDF sensors are strained. Due to the piezoelectric properties, a small voltage is generated. If the small animal under study is 2–3 cm apart between the front legs, it is easily detected in which unit sensor it is positioned. Through the weight–pressure triangulation relationship, the free animal’s exact direction and orientation are recognized. That relative position is given to a control unit responsible for controlling three minimal noiseless mechanized servo systems (3 axes of possible movement), designed to maintain the optical receiver above the head of a moving animal. The optical receiver is composed of a 2 cm by 2 cm photodetector array, ensuring accurate light beam alignment in a free-behavior animal for high-transmission data rates. The optical receiver unit was placed 50 mm above the animal. The 4 cm^2^ photodetector array allows a large active area covering the transmitter’s optical beam exposure and allows the free behavior of animals. Figure 3 shows the real concept of the CPFT.

To validate the concept presented in Figure 1 and Figure 2, an OWCS based on a wavelength of 850 nm for data transfer was demonstrated in optical tissue phantoms (OTPs) with different thicknesses (2 mm, 5 mm, and 10 mm) and including an air-free space of 50 mm. An optical transmitter (with a LED) and an optical receiver (with a photodiode) were used to establish the optical link, and all the relevant considerations to mimic the conditions presented in Figure 1 and Figure 2. The baud rate values were calculated for several optical transmitter powers and considering the three different tissue phantoms. The analysis of the signal format inherent to the optical link was also performed. At last, the error signal rates were obtained, also considering the three different tissue phantoms.

## 2. Materials and Methods

### 2.1. Optical Wireless Communication System (OWCS)

The OWCS has two parts: the optical transmitter, which can be inside of a small animal, e.g., integrated into an invasive neural probe [18], and an optical receiver, which can be in a cage ceiling.

The acquired data from the small animal reaching the receiver device must be encoded following a specific communication protocol. The OWCS is able to send universal asynchronous receiver/transmitter (UART) logical data using intensity modulation with direct detection (IM/DD) [19]. This method directly converts the light intensity into an electrical current. This signal will undergo elaborate data processing.

The on-off keying (OOK) modulation was implemented by switching the optical source “on” or “off”, corresponding to serial binary 1’s or 0’s. Therefore, the “0” bit corresponds to the absence of light and the “1” bit corresponds to the presence of light during bit pulses. For logical levels used to identify serial data, binary “1” was identified by a voltage around 3.3 V, and the binary “0” was identified by a voltage around 0 V. This range can be adjusted to the range between 0 V to 1.5 V, reducing the system power consumption.

The goal of the transmitter block consists of converting the UART logical data into the LED current (i.e., different logical levels correspond to different levels of current transmitted) according to modulation standards. For that purpose, a specific metal oxide semiconductor field effect transistor (MOSFET) was used as a switch to control the LED behavior. The MOSFET model ZVN3310FTA from Diodes Incorporated, Texas, USA [20] was also used. A high-speed LED from Vishay, Pennsylvania, USA, model VSLY5850 [21], based on an AlGaAs surface was also used. This LED presents an emission peak centered at 850 nm, with a fall/rise time of 10 ns. Furthermore, this LED has an angle of half intensity of 3 degrees, reducing power losses by tissue scattering and absorption. By controlling the current for values near 70 mA, it is possible to obtain an optical power of approximately 55 mW. The option LED versus VCSEL (vertical-cavity surface-emitting laser) is based on the high-optical power to allow optical telemetry communication on the deeper tissues, instead of the faster switching speed and low-power consumption of the VCSELs. The OWCS works continuously for approximately 2 h using a coin Zinc/Silver oxide battery cell model 399 SR927W from Energizer Company, Missouri, USA with a rated capacity of 55 mAh.

The receiver block is responsible for transforming the relative differences of light intensity into UART logical binary signals for later software processing and establishing optical communication between the transmitter and the receiver. The receiver block contains a photodetector extremely sensitive to 850 nm light, with high responsivity (approximately 0.52 A/W at 850 nm) for minimum light changes made by the transmitter. A photodiode model PS11.9-5 TO from First Sensor Company, Berlin, Germany [22] was selected with an active area of 11.9 mm^2^ and a rise time of 3 ns. In the final design (Figure 2), an array of photodiodes with a large square active area of 4 cm^2^ must be considered. A transimpedance amplifier (TIA) model AD8015 from Analog Devices, Massachusetts, USA was used (bandwidth of 240 MHz) [23]. Also, a comparator (model AD8561 from Analog Devices, Massachusetts, USA [24]) was used to ensure the UART logic values with a high-speed propagation time of 7 ns, digital output (0–3.5 V), with a fall time of 1.5 ns and rise time of 3.8 ns.

**Figure 4:**
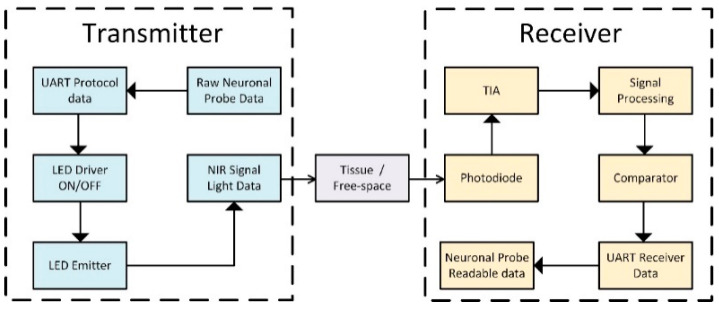
OWCS block diagram.

Figure 4 illustrates the complete OWCS block diagram.

### 2.2. Optical Tissue Phantoms (OTPs)

The validation of the OWCS was performed on OTPs with specific optical properties that are durable and stable in time [25,26,27]. The OTPs simulate the animal tissue and were made from a bulk material with tunable scattering and absorbing coefficients. The OTPs bulk material is based on 200 mL polydimethylsiloxane (PDMS) with 20 mL of curing agent and presents a refractive index of 1.4 [26]. The OTPs scattering is obtained through 0.16 mg of alumina (Al_2_O_3_) particles, previously filtered in sizes between 25 µm to 75 µm. Al_2_O_3_ has a high scattering coefficient, is inexpensive, and provides a reasonably reliable means to mix a scatterer into a solution [27]. The absorption is obtained with 0.56 mg of India ink. The scattering and absorption agents were mixed with the OTPs bulk material solution for 20 min and the air bubbles were removed in a vacuum chamber for 1 h. The last process step consisted of curing the OTPs solution for 1 h in an oven at 100 °C. The developed OTPs were characterized by a monochromator model 260 1/4 M from Oriel Cornerstone, Wales, United Kingdom, at a fixed wavelength of 850 nm and a homogeneous attenuation coefficient of 1.03 cm^−1^ was obtained by a power meter model 1918 R from Newport, Wales United Kingdom. The developed OTPs have three different thicknesses, 2 mm, 5 mm, and 10 mm.

**Figure 5:**
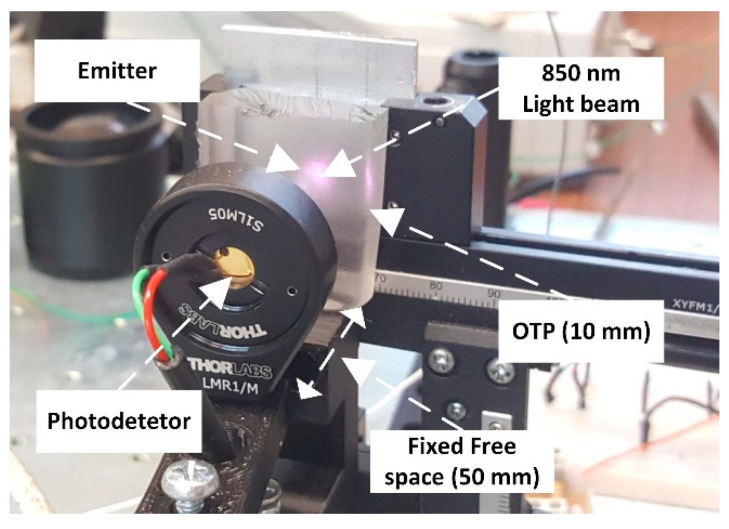
Experimental setup to evaluate the OWCS with a developed OTP.

Figure 5 shows the implemented experimental setup to evaluate the OWCS, considering an optical tissue phantom (OTP) with 10 mm thickness.

A binary sequence of bits was sent to the transmitter block by the computer, according to the predefined communication protocol. The LED was mounted on a 3-axis millimetric precision translation stage. Each OTP was placed with a precise alignment between the LED and the photodetector. Also, each OTP was in close contact with the LED (without compressing it) and with a gap of 50 mm from the photodetector (the air-free space). Thus, the distance between the LED and the photodetector was 50 mm plus the OTP thickness.

## 3. Results and Discussion

To evaluate the OWCS, the measurements were divided into three main parts: (a) maximum data rate capable of being transmitted with low-power consumption; (b) analysis of the signal format inherent to the optical link; and (c) error signal rates.

Reliable optical telemetry was defined for a predefined baud rate if the system received at least 95% of the sent data without errors.

**Figure 6:**
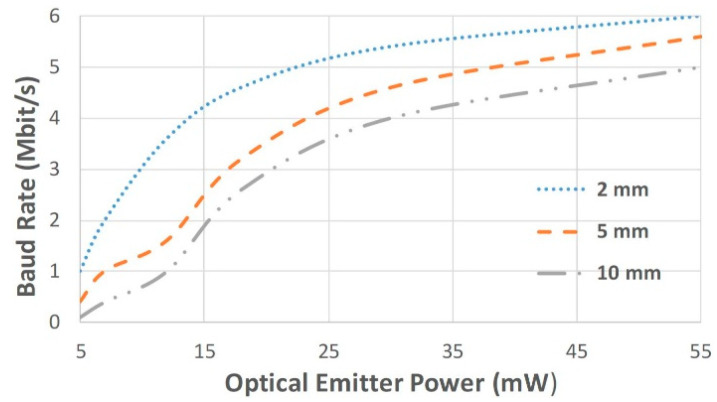
Relationship between OWCS optical emitter power and baud rate for different OTPs thicknesses.

Figure 6 shows the OWCS results as the relationship between the achieved maximum speed and the LED optical power used for the different OTPs thicknesses (2 mm, 5 mm, and 10 mm). Also, a fixed 50 mm free space is considered between the OTP and the detector to mimic a real scenario, as the one presented in Figure 2.

The high-speed data telemetry was achieved as the OWCS reached a maximum baud rate of 6 Mbit/s, 5.5 Mbit/s, and 5 Mbit/s, using 55 mW of optical power (70 mA of electric current), for 2 mm, 5 mm, and 10 mm OTP thick, respectively. The baud rate of 2 Mbit/s required optical power of around 15 mW with 10 mm OTP thick. At 15 mW of optical power (around 20 mA of electric current), baud rates of 2.5 Mbit/s and 4.2 Mbit/s for 5 mm and 2 mm are obtained, respectively. At 35 mW of optical emitter power (approximately 55 mA of electric current), the OWCS allows communication higher than 4 Mbit/s for all 3 different OTPs thicknesses. Therefore, the OWCS can be used in in-body devices for high transmission data rates at reasonable energy disbursements. The increasing of the baud rate requires the increasing of the optical emitter power. These results are explained by the necessity of achieving a certain baud rate with a certain optical power threshold to cross an OTP thickness. Also, it is observed that after the optical light beam transposes a certain thickness, it becomes easier to achieve high transmission data rates. As higher the intensity of the light beam, the easier becomes to reach high-speed telemetry and calibrate the receiver block.

The analysis of the received signal was studied in detail, including maximum and minimum signal voltages, noise, rise and fall times, and total transmission latency. A stable optical link was observed, with the received (Rx) signal being very similar to the transmitted one (Tx). Also, it is noted that the Rx signal is well restricted to two logical values, with 3.5 V and 0 V to the logical levels “1” and “0”, respectively. These values agree with the predefined UART protocol logical levels. For the existing signal noise, a random and non-significant noise (with mV scale) was found, not affecting the receiver decision between logical values. Moreover, the Rx signal shows fall and rise times of 3.5 ns and 8.5 ns, respectively, and a total transmission latency of 100 ns.

**Figure 7:**
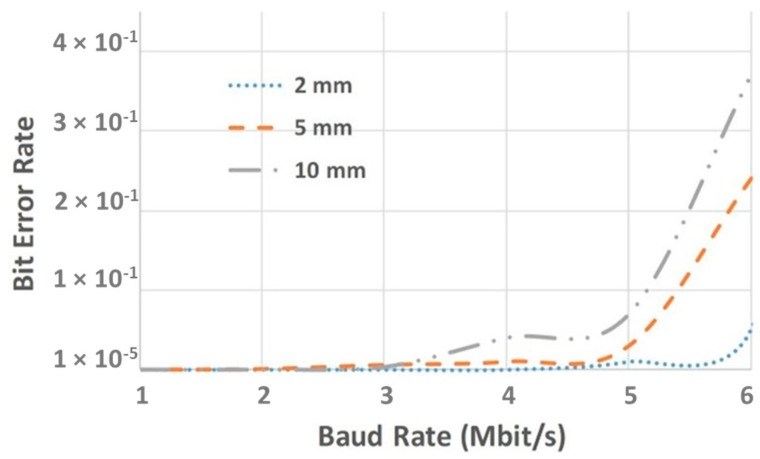
OWCS BER for different baud rates using 55 mW of optical power.

Finally, the Bit Error Rate (BER) was studied. A known random sequence pattern was sent by the emitter to the receiver. After the complete transmission of the pattern, a software script was developed to compare the received pattern with the emitted known pattern. The developed script counts the number of bits and checks the position for each bit. Figure 7 shows the BER metric for 2 mm, 5 mm and 10 mm of OTP thickness crossed using 55 mW of optical power. It was observed that for baud rates up to 3 Mbit/s, a BER of 1 × 10^−5^ for all different tissue thicknesses was obtained. For baud rates above 4 Mbit/s, the BER rates increase for thicker OTP due to more significant beam spreading, scattering, and absorption coefficients. At 5 Mbit/s, the OWCS achieved a BER of 1 × 10^−2^, 2 × 10^−2,^ and 7 × 10^−2^, for 2 mm, 5 mm, and 10 mm of OTPs crossed, respectively. These results show a well-defined optical communication link that can sustain reliable and clean high data rates up to 6 Mbit/s through an OTP thickness of 2 mm with a BER of 5 × 10^−2^ at 55 mW of optical power.

Table 2 summarizes the performance of the developed OWCS in comparison with some literature studies in optical telemetry systems centered at 850 nm. This OWCS is able to achieve high-transmission data rate optical telemetry (>2 Mbit/s) through a thicker tissue (approximately 10 mm) and an air-free space of 50 mm, maintaining a reasonable optical power consuming (maximum of 55 mW) and an implementation with optical components suitable for small animal physiology telemetry.

Vertical-cavity surface-emitting lasers (VCSELs) and LEDs can be used to establish optical wireless communication systems. Laser sources allow high-speed communications, but they can have more harmful effects (e.g., for eye retina) because they are point-source emitters. LED technology can be used due to its cost-effectiveness, safety, and wide bandwidth. Furthermore, LEDs have a long lifetime due to their more resistant materials, and license-free operation [28,29,30].

Following the features previously described, this study has successfully implemented and validated an optical communication system based on a standard LED and photodiode, mimicking a real scenario of data transfer from an animal with a neural probe for brain activity recording.

**Table 2:** Comparison of the developed OWCS and optical telemetry systems in the literature.

Wavelength (nm)	Optical Power (mW)	Link Speed (Mbit/s)	Emitter Type	Receiver Type	Thickness (mm)	Free-Space (mm)	Ref.
850	4.3	40	VCSEL	Photodiode	3	-	[31]
850	7.5 10 20	16	VCSEL	Photodiode	2 4 6	-	[32]
850	2.6 4.1 6.4	50	VCSEL	Photodiode	2 4 6	-	[33]
850	15 35 55	2 4.2 5	LED	Photodiode	10	50	This work

## 4. Conclusions

This paper describes the design of an OWCS for transmission of data. An OWCS based on a wavelength of 850 nm, with a data transfer of 5 Mbit/s, optical power of 55 mW, for a tissue phantom thickness of around 10 mm, and an air-free space of 50 mm was developed. It was concluded that the OWCS can achieve high-transmission data rate optical telemetry (>2 Mbit/s), compared to standard RF wireless communication for in-body devices. This OWCS integrated into a neural microsystem (e.g., invasive neural probe) can provide continuous neural measurements and transmission (at high-transmission data rates) in behavior animal experiments. Moreover, the use of the OWCS with a CPFT will allow automatic tracking of the position of the small animal. The CPFT is a very low-cost solution that allows us to obtain in real-time the exact position of the optical transmitter implanted in the small animal, allowing the alignment between the optical transmitter and the optical receiver to ensure a reliable optical link.

## Figures and Tables

**Table 1:** Summary of the materials used to design LEDs with different wavelengths [13].

Wavelength (nm)	Material
440–550	InGaN
570–650	AlGaInP
624–920	AlGaAs

## Data Availability

Data are contained within the article.
